# Efficient High-Power Ultrashort Pulse Compression in Self-Defocusing Bulk Media

**DOI:** 10.1038/s41598-017-01504-x

**Published:** 2017-05-03

**Authors:** Marcus Seidel, Jonathan Brons, Gunnar Arisholm, Kilian Fritsch, Vladimir Pervak, Oleg Pronin

**Affiliations:** 1 Max-Planck-Institut für Quantenoptik, Hans-Kopfermann-Str. 1, D-85748 Garching, Germany; 2 UltraFast Innovations GmbH, Am Coulombwall 1, D-85748 Garching, Germany; 3 Ludwig-Maximilians-Universität München, Am Coulombwall 1, D-85748 Garching, Germany; 4 FFI (Norwegian Defence Research Establishment), P. O. Box 25, NO-2027 Kjeller, Norway

## Abstract

Peak and average power scalability is the key feature of advancing femtosecond laser technology. Today, near-infrared light sources are capable of providing hundreds of Watts of average power. These sources, however, scarcely deliver pulses shorter than 100 fs which are, for instance, highly beneficial for frequency conversion to the extreme ultraviolet or to the mid- infrared. Therefore, the development of power scalable pulse compression schemes is still an ongoing quest. This article presents the compression of 90 W average power, 190 fs pulses to 70 W, 30 fs. An increase in peak power from 18 MW to 60 MW is achieved. The compression scheme is based on cascaded phase-mismatched quadratic nonlinearities in BBO crystals. In addition to the experimental results, simulations are presented which compare spatially resolved spectra of pulses spectrally broadened in self-focusing and self-defocusing media, respectively. It is demonstrated that balancing self- defocusing and Gaussian beam convergence results in an efficient, power-scalable spectral broadening mechanism in bulk material.

## Introduction

Many important breakthroughs in ultrafast optics during the past thirty years have been strongly connected to the emergence of titanium doped sapphire (Ti:Sa) crystals as the active laser medium in femtosecond (fs) technology. The development of attosecond physics^1^ as well as frequency comb spectroscopy^2^ are two striking examples. Moreover, many femtochemistry laboratories rely on this solid-state laser architecture^3^. The attractiveness of the Ti:Sa technology stems, in particular, from the ultrabroadband emission bandwidth of the gain material^4, 5^. With proper dispersion control, it readily enabled the generation of few-cycle pulses, a prerequisite for field-sensitive nonlinear optics^6^, self-referencing schemes for the stabilisation of an optical frequency comb^7^ and a high selectivity of electronic transitions in various molecular samples.

However, the Ti:Sa technology exhibits only limited power scalability. This is due to the lack of available high power pump diodes in the green as well as detrimental nonlinear and thermal effects in the rod-type gain materials (cf. e.g. ref. 8). As peak power triggers nonlinear effects like frequency conversion or multi-photon ionization while laser repetition rate determines the data acquisition rate, the combination of both is needed in various experiments, for example, in extreme ultraviolet (XUV) and mid-infrared (mid-IR) frequency comb spectroscopy^9, 10^, time-resolved photo-emission electron microscopy^11^ or coincidence spectroscopy^12^. Therefore, simultaneous scaling of peak and average power presents the key point of current femtosecond technology developments^13^.

The most powerful laser architectures, namely (thin-)disk^13, 14^, innoslab^15^ and fibre^16^ technologies, are mainly based on Yb-ion doped host materials which exhibit exceptional thermal properties and can be directly pumped with InGaAs laser diodes. However, the fluorescence linewidth of Yb:YAG is, for instance, only about Δλ_f_ = 9 nm full width at half of the maximum (FWHM) at room temperature^17^, compared to Δλ_f  _= 230 nm for Ti:Sa^18^. This points out the general difficulty of the Yb-based lasers to directly emit sub-100 fs pulses and highlights the need for power-scalable ultrashort pulse generation schemes.

Beyond that, Krausz *et al*.^4^ and French^5^ explain that the success of the Ti:Sa technology was also caused by rather practical advantages over previous architectures, namely low cost, low complexity and high reliability. Usually, amplification-free systems come with these properties, and thus the development of mode-locked thin-disk (TD) laser oscillators has been subject to intense research since their first demonstration in the year 2000^19^. Today, these fs laser oscillators deliver average powers of more than 250 W^20, 21^, pulse energies of up to 80 *μ*J^22^ and peak powers of more than 60 MW^22, 23^. For comparison, the power limits of today’s Ti:Sa oscillators may be represented by the results of ref. 8. Those are 2.5 W average power, 0.5 *μ*J and 10 MW peak power. Despite the successful efforts in power scaling, mode-locked oscillators may never reach the peak powers of kHz amplifier systems. Consequently, peak power increase through pulse compression becomes a prerequisite to efficiently drive strong-field effects like high harmonic generation.

Low complexity and high scalability were also important salient points when compression of ultrashort pulses after spectral broadening in a bulk material was introduced in 1988^24^. However, an efficiency of only 4% was reached for a pulse compression factor of 5. High losses are inherent to the propagation of an intense Gaussian beam in a long Kerr medium if the peak power of the ultrashort pulses clearly exceeds the critical power of the material. Milosevic *et al*. pointed out that self-focusing causes the excitation of higher order spatial modes which transfer into losses after pulse cleaning^25^. It was explained that guided waves overcome this issue and briefly noted that multi-pass geometries may reduce spatial losses for low peak powers. This multi-pass or multi-plate approach has been extended to a huge peak power range by now^26–29^. It exhibits efficiencies of at least 40%.

This report demonstrates a different approach to efficient ultrashort pulse compression in bulk material. It is based on a combination of Gaussian beam convergence and self-defocusing. Cascaded quadratic (*χ* ^(2)^) nonlinearities in beta barium borate (BBO, *β*-BaB_2_O_4_) crystals are exploited for this purpose. They give rise to an optical Kerr-like effect whose sign and magnitude depends on the phase-mismatch of the underlying three-wave mixing process, i.e. second harmonic generation (SHG)^30, 31^. In the experimental part of the report, the compression of initially 180 fs pulses to 30 fs is demonstrated at a 70 W average power level and with 75% efficiency. The following simulation part will explain why the combination of self-defocusing nonlinearities and Gaussian beam divergence can lead to an efficient spectral broadening mechanism. The demonstrated concept may also be applied to a variety of other high power ultrashort pulse light sources.

## Results

### Setup

**Figure 1:**
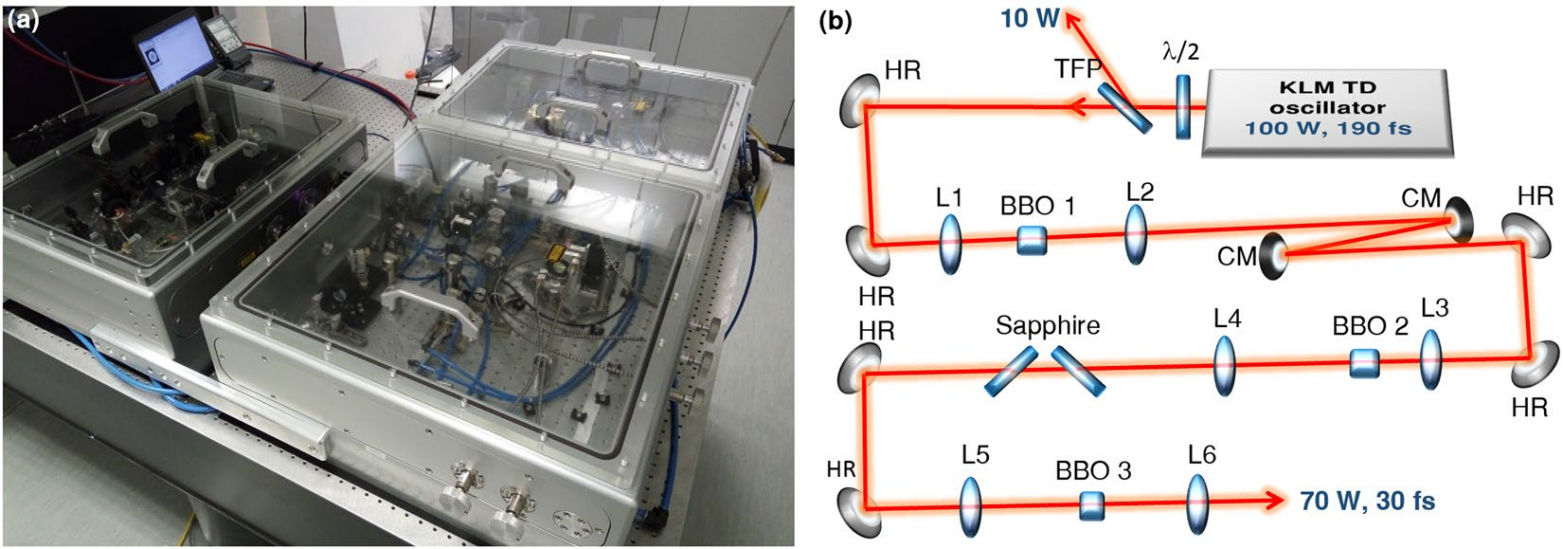
(**a**) Photo of oscillator and compression chamber. The larger housing contains the KLM TD oscillator. The smaller housing contains the compression setup which is sketched in (**b**) The power sent to the BBO crystals was controlled by the half-wave plate (*λ*/2) and the thin-film polarizer (TFP). The beam was steered by pairs of 45° highly reflective dielectric mirrors (HR). All lenses (L1-L6) were plano-convex, anti-reflection (AR) coated fused-silica lenses. The chirped mirrors (CM) exhibited a group delay dispersion (GDD) of +2000 fs^2^. The two sapphire plates were 5 mm thick and placed at Brewster’s angle. The BBO crystals were mounted on a translation stage and could be rotated in the vertical direction for the purpose of angle-tuning. BBO 1 and BBO 2 were 10 mm long and BBO 3 6 mm. The apertures were 7 × 7 mm^2^.

The pulses entering the compression setup emerged from a commercial-grade Kerr-lens mode-locked (KLM) TD oscillator (UltraFast Innovations GmbH). It was set up in a monolithic aluminium housing which had a footprint of 145 cm × 70 cm. The housing itself and all optics mounts inside were water-cooled. Moreover, the oscillator could be aligned without opening the housing. This allowed stable operation (power RMS ≈ 0.5%, calculated from 5000 samples, 1 sample/s). The oscillator delivered 190 fs sech ^2^-pulses centred at about 1030 nm with 4.2 *μ*J energy at a repetition rate of 23.8 MHz. This corresponds to an average power of about 100 W. The oscillator was set up according to the principles described in ref. 21. A photograph of the laser is shown in Fig. 1(a).

The compression chamber consisted also of a monolithic, water-cooled housing. It had a footprint of 70 cm × 55 cm and contained three sequential pulse compression stages. The setup is sketched in Fig. 1(b). The crystals were water-cooled. Their temperature was between 25 and 30 °C in thermal equilibrium at 90 W input power. The spectral broadening was achieved in BBO crystals by virtue of cascaded phase-mismatched *χ* ^(2)^-nonlinearities, resulting in an effective negative nonlinear refractive index which can be expressed as^30^:

1 $${n}_{2}(\theta ,\lambda )={n}_{2}^{({\rm{Kerr}})}+{n}_{2}^{({\rm{cas}})}(\theta ,\lambda ),$$

where ${n}_{2}^{({\rm{Kerr}})}$ is the positive nonlinear refractive index resulting from the optical Kerr-effect and ${n}_{2}^{({\rm{cas}})}$ (*λ*, *θ*) describes a nonlinear refractive index-like term arising from the quadratic nonlinearities of BBO. It can be varied in magnitude and sign via tuning of the crystal angle *θ*, i.e. the phase-matching of the incoming beam and its second harmonic. Moreover, it exhibits a much stronger wavelength (*λ*) dependence than the Kerr effect near the phase-matching angle for SHG. Supplement 1 shows additional details on how angle-tuning of the nonlinear crystals manipulates magnitude and dispersion of *n*_2_(*θ*, *λ*).

### Pulse compression and beam quality

**Figure 2:**
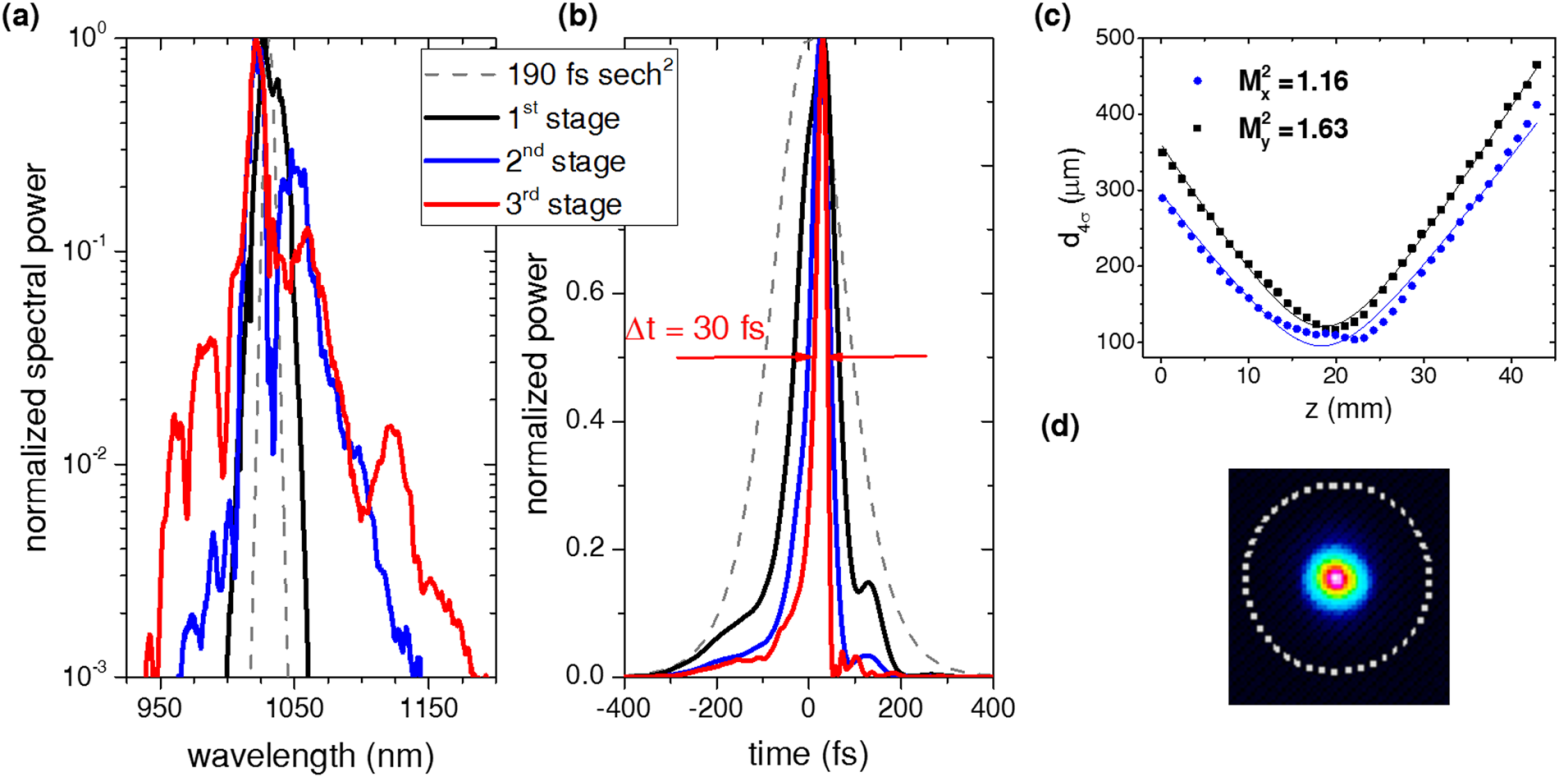
(**a**) Broadened spectra measured with an OSA. (**b**) Retrieved FROG traces with 97 fs (black line), 50 fs (blue line), 30 fs (red line) full widths at half maximum. The legend applies to (**a**,**b**). As a reference a sech^2^-shape spectrum and pulse corresponding to the oscillator input is shown. (**c**) M^2^ measurement of the beam behind the third broadening stage. (**d**) Focused beam profile extracted from the M^2^ measurement shown in (c).

About 90 W were focused with a 60 mm focal length lens into a 10 mm long BBO crystal whose front facet was placed about 50 mm behind the lens. The broadened spectrum was compressed by two bounces off chirped mirrors with +2000 fs^2^ GDD. The compression factor was adjusted to about 2 which results in low power in the pulse pedestals if only first order chirp is compensated^28^. In the following stage, the pulses were focused with an *f* = 50 mm lens into another 10 mm BBO crystal. Since the absolute value of GDD required for pulse compression decreases with increasing bandwidth, utilizing the normal dispersion of two 5 mm thick sapphire plates was sufficient to reduce the pulse duration to about 50 fs. In the final stage, an *f* = 100 mm lens and a 6 mm BBO were utilized. No additional dispersive optics for pulse compression were needed because the positive group velocity dispersion (≈48 fs^2^/mm at 1030 nm) in combination with the negative nonlinear phase shift self-compressed the pulses to about 30 fs. The spectra, measured with an optical spectrum analyser (OSA), and the pulses, retrieved by second-harmonic frequency resolved optical gating (FROG) measurements, are shown in Fig. 2. The compression results are also summarized in Table 1. After the third broadening stage, the red wing of the spectrum was close to the second harmonic resonance. This could be observed through the emergence of weak, visible red radiation. It is inferred that further spectral broadening would require a stronger detuning from the second harmonic phase-matching angle, and thus a reduced magnitude of the defocusing nonlinearity. Scanning the far-field beam profile with a multimode fibre connected to an OSA revealed very good beam homogeneity. This was experimentally also confirmed by the good agreement between the spectrum measured with the OSA and the one retrieved from FROG (cf. supplement 2). About 70 W of average power were emerging from the third BBO. The remaining 20 W were partly converted into the second harmonic (about 2% per stage) which was mainly transmitted through the 45° HR mirrors. The majority of the losses arose from the 30 interfaces the beam had to pass. Each stage exhibited an efficiency between 90 and 93%. This resulted in an efficiency of more than 75% and a compression factor of more than 6 which is akin to fibre broadening results. The polarization extinction ratio was better than 1:30. A peak power of 60 MW was reached. It might be further increased to 75 MW by using tailored chirped mirrors (cf. supplement 2).

**Table 1:** Summary of the presented pulse compression experiments.

	Fourier transform limit	added GDD^*a*^	measured pulse duration	peak power
1st stage ^*b*^	82 fs	4000 fs^2^	97 fs	27 MW
2nd stage	50 fs	360 fs^2^	50 fs	45 MW
3rd stage	25 fs	0 fs^2^	30 fs	60 MW

^*a*^An additional GDD of ≈80 fs^2^ is added by the collimation lens after each stage.

^*b*^For comparison, the simulation of the 1^st^ stage yielding the spectrally resolved profile shown in Fig. 3(c) resulted in a 74 fs Fourier transform limit.

In addition to the characterization of the pulses, an M^2^ measurement in accordance to the ISO Standard 11146 was performed. The M^2^ factor in horizontal direction was ${M}_{h}^{2}=1.2$ and clearly better than that in vertical direction ${M}_{v}^{2}=1.6$ (Fig. 2(c)). This is attributed to spatial walk-off in the birefringent crystals. Nevertheless, the beam can be focused well as Fig. 2(d) shows. Most remarkable, no self-diffraction rings like observed in positive *n*_2_-based spectral broadening^28^ were detected.

### Simulations investigating spatial properties

In ref. 28 simulations showed that firstly, spatial beam inhomogeneity increases with peak power if a material’s critical power is exceeded. Secondly, compensating self-focusing by Gaussian beam divergence leads to an even stronger inhomogeneity of the spectral broadening across the beam diameter. Only a fraction of the beam is trapped in a region of high intensity where self-phase modulation (SPM) happens, while most of the light is not captured and hence does not undergo spectral broadening. This effect is illustrated by means of a crude model, adapted from R.Y. Chiao *et al*.^32, 33^, which is presented as supplement 3. If, by contrast, a combination of self-*de*focusing and Gaussian beam *convergence* is applied, it is expected that the effect can be reversed, i.e. that the beam gets homogeneously broadened.

**Figure 3:**
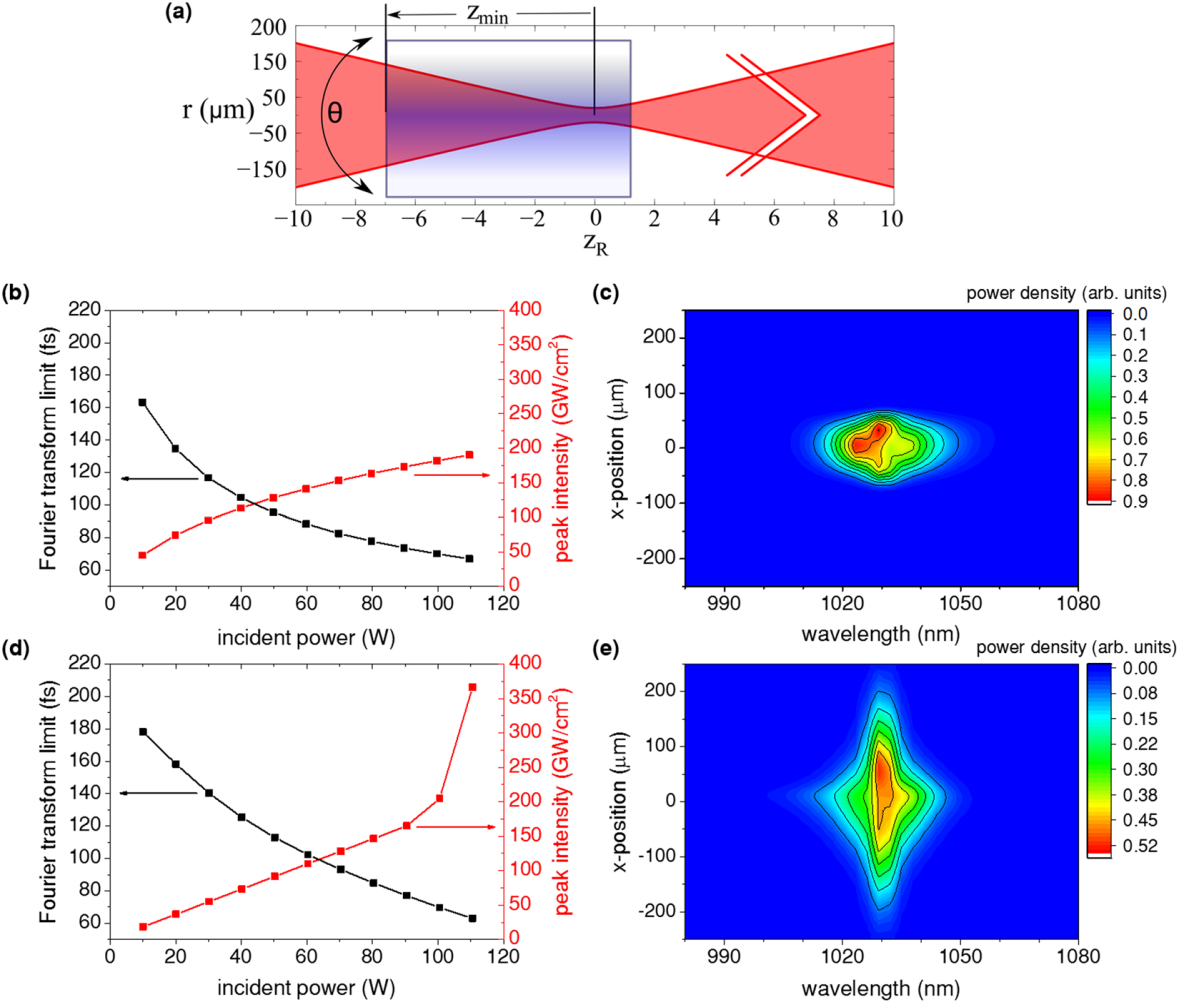
Simulations with an incoming 180 fs sech^2^-pulse, focused to a beam diameter of 40 *μ*m (in absence of nonlinear refraction): (**a**) Sketch of the simulation parameters. The tuning angle *θ* was varied to achieve self-defocusing and -focusing, resp. The effective nonlinear refractive indices were ${n}_{2}=\mp 8\cdot {10}^{-16}\,{{\rm{cm}}}^{2}/{\rm{W}}$. They resulted from the sum of Kerr and cascaded *χ* ^(2)^ effects in both cases. A hypothetical ${n}_{2}^{({\rm{Kerr}})}$ = −4 · 10^−16^ cm^2^/W was set in the self-focusing case to match the magnitudes of the contributions to *n*_2_. The distance from the crystal front facet to the focal plane was *z*_min_ = −7*z*_*R*_ (−8.5 mm, illustrated in the figure) in the defocusing and *z*_min_ = 4*z*_*R*_ (4.9 mm) in the focusing case. This has been chosen to match Fourier transform limit and peak intensity at 90 W incident power. The Rayleigh length *z*_*R*_ was about 1.22 mm, the crystal length 10 mm. The beam radius is denoted by r. The red arrows indicate the propagation direction (z-axis). (**b**) Dependence of the maximal peak intensity inside the crystal and the Fourier transform limit of the pulses on the incident power for *negative* effective *n*_2_ and beam *con*vergence. (**c**) Spectral power distribution in dependence on the x-position with respect to the beam centre for *negative* effective *n*_2_ and beam *con*vergence. (**d**) Dependence of the maximal peak intensity inside the crystal and the Fourier transform limit of the pulses on the incident power for *positive* effective *n*_2_ and beam *di*vergence. (**e**) Spectral power distribution in dependence on the x-position with respect to the beam centre for *positive* effective *n*_2_ and beam *di*vergence. (**c**,**e**) show line-outs at y = 0 *μ*m for 90 W incident power.

The simulation tool^34^ that has been used to investigate the coupling of spatial and spectral nonlinear effects in ref. 28 is employed to highlight the favourable beam properties which were accomplished by exploiting self-defocusing nonlinearities, i.e. negative effective *n*_2_. As an example, the spectral broadening of the first compression stage is studied. Figure 3 compares the case of a self-defocusing nonliearity and beam convergence ((b) and (c)) with self-focusing in combination with beam divergence ((d) and (e)). Convergence and divergence refer to the front facet of the nonlinear crystal which is displaced from the focal plane by *z*_min_ (Fig. 3(a)). Beam convergence, peak intensity at the BBO entrance facet and crystal length in the self-defocusing case are similar to the experimental conditions. For an incident power of 90 W a Fourier transform limit of about 75 fs is reached for both the simulation of positive and negative effective *n*_2_. This is also comparable to the experimental result (cf. Table 1). A first significant difference is the dependence of the maximal peak intensity inside the crystal on the incident power. In the self-defocusing case, the peak intensity rises sub-linearly (Fig. 3(b)). On contrary to this, the intensity increases first linearly in the positive *n*_2_ case (Fig. 3(d)), but at about 100 W of average power rapidly rises, indicating beam collapse which would ultimately lead to crystal damage. The soft optical-limiting behaviour in the self-defocusing case is favourable since it makes the broadening less susceptible to intensity fluctuations and more robust against damage. The most crucial difference becomes apparent in Fig. 3(c,e). While in the self-focusing case only the central part is spectrally broadened and the incident spectrum around 1030 nm is spread out in space, in the self-defocusing case such a wavelength dependent spatial distribution is not observed, but the whole beam is spectrally broadened. In both cases, the influence of spatial walk-off becomes visible which is a disadvantage of critically phase-matching the birefringent crystals. It is a cause for the increase of the M^2^ factor after the compression stages. Nevertheless, the simulation results clearly point out the advantages of combining self-defocusing with beam convergence and explain the efficiency of the experimentally realized compression scheme. The sensitivity of the spectral broadening on the variation of the distance between focal point and crystal position as well as on the tuning angle was also studied by means of simulations. The results are provided in supplement 4.

## Discussion

The initial experiments on spectral broadening in BBO already pointed out in a brief statement that nonlinear beam distortions became only visible in the self-focusing regime^31^. However, these experiments were conducted with a Ti:Sa based laser system, operating at much lower average power but about 4.7 GW peak power. Hence, the 17 mm long BBO crystal could be placed in a collimated beam, and thus the Rayleigh range clearly exceeded the crystal length. Beam distortions in the self-defocusing regime became apparent and were explicitly stated in experiments with about 100 MW peak power^35^, i.e. in a peak power range where the latest generation of mode-locked TD oscillators operates. The issue was addressed by utilizing flattop beams which do not exhibit a continuous spatial gradient and therefore should be homogeneously spectrally broadened^36^. Although the beam homogeneity improved, adding a beam shaper also added complexity to the setup and introduced losses of about 30%. Moreover, beam shaping will be complicated owing to the average powers on the order of 100 W. Therefore, the proposed method of combining beam convergence and nonlinear self-defocusing presents a novel, elegant alternative to achieve efficient pulse compression in bulk material.

The fact that the initial experiments performed with several GWs of peak power did not reveal beam distortions, implies the peak power scalability of the approach. It is expected that the scheme even benefits from higher peak powers since the beam would have to be focused less tightly, and thus the nonlinear defocusing can be reduced. Consequently, the phase-mismatch can be increased and the compression scheme can support bandwidths which allow few-cycle pulse generation^35^. To the best of the authors’ knowledge, the cascaded quadratic nonlinearities have been employed for the first time in pulse compression of a Watt-class laser source although this has been proposed more than six years ago for high power (fibre-based) lasers with pulse parameters comparable to those of the utilized TD oscillator^37^. It is to note that the achieved pulse duration of 30 fs goes beyond the predictions of those earlier studies because the simulations of ref. 37 aimed for self-compression in a single crystal while the presented experiments targeted a compression factor of two in each of the three stages.

Pulse compression of high power laser systems has also been performed with gas-filled (Kagomè-type) hollow-core photonic crystal fibers (HC-PCFs)^38–40^ which offer broadband guiding and tight confinement of the laser beam to radii on the order of tens of microns. For peak powers of at least several hundreds of MWs, which can be reached by amplifier systems with hundreds of Watts average power, gas-filled capillaries have been utilized as well^41^. Inert gases exhibit even at high pressures very low group velocity dispersion as well as GW-level critical powers (depending on the gas pressure)^42^. Therefore, fibres usually allow large broadening factors. They are also well-suited for generating few-cycle pulses at up to GWs of peak power^6^ and often exhibit power efficiencies of 70–80%. However, bulk broadening schemes have many practical advantages over fibre-based approaches. They do not require sensitive coupling of the free beam mode to the fibre mode. They are compact, robust and cost-efficient. The large apertures allow to shift the crystals after occurrence of damage without the need to replace them if heat accumulation is avoided. In our own studies^28, 39^, we also experienced much better noise figures of bulk broadening and we encountered detrimental ionization near the input facets of the Kagomè-type HC-PCFs which was not expected from kHz experiments and required employing positive gas pressure gradients. If the bulk broadening technique presented here, is compared to Kerr-effect-based approaches^24, 28, 43, 44^, it shares the just mentioned advantages over fibre-based methods. In addition, the compression scheme presented here exhibits efficiencies comparable to fibre. Furthermore, the scheme allows to use dielectric materials to compensate chirp or even to achieve self-compression. This has been studied for cascaded quadratic nonlinearities of BBO in more detail in refs 35, 45. Utilizing the cascaded *χ* ^(2)^-effect exhibited also a few drawbacks: Firstly, the M^2^ factor in the direction of the extraordinary crystal axes increased from 1.1 to more than 1.6. It is expected to improve if the crystals are cut for normal incidence, i.e. at *θ* = 21.5° instead of *θ* = 23.5°. However, the spatial walk-off is intrinsic due to the need for critical phase-matching for BBO. If periodically poled nonlinear crystals like LiNbO_3_ (PPLN) or KTP are used, the problem of walk-off could be avoided. Pulse compression in PPLN was predicted for 100 fs, nJ-level pulses at 1550 nm^46^ (type 0 phase-matching) and experimentally demonstrated for 110 fs, 30 *μ*J pulses at 1560 nm (type 1 phase-matching)^47^. For more energetic pulses, LBO presents also an alternative to BBO. The walk-off at room temperature is about a factor 7 smaller and non-critical phase-(mis-)matching at 1030 nm can be achieved by heating the crystal^48^. But the quadratic nonlinearity is also reduced by 60% in comparison to BBO and the dispersion of negative effective *n*_2_ is stronger at 1030 nm. Furthermore, tandem crystal geometries like employed in optical parametric amplifiers or oscillators^49^ could reduce the spatial walk-off in the setup. A second drawback is the relatively complex initial alignment owing to the interplay of crystal angle, crystal length and crystal position with respect to the focal point and spot size. Thirdly, the cascaded *χ* ^(2)^-nonlinearity is dispersive, and thus the generation of few-cycle pulses may require to reduce the absolute value of the effective *n*_2_^35^ or the combination with Kerr-effect based bulk broadening in a multi-plate assembly^27, 28^ or multi-pass cell^29^. However, this also strongly depends on central wavelength and pulse energy as few-cycle pulse generation has already been shown for different laser parameters^35, 50, 51^.

In summary, spectral broadening based on cascaded *χ* ^(2)^-nonlinearities was performed at unprecedented high average power levels of 90 W. The previously reported experiments were done at kHz repetition rates and high average power applications were only subject to simulations^37^. An increase in peak power from 18 MW to 60 MW and the generation of 30 fs pulses makes the source well-suited for high-power mid-infrared generation^52^. Moreover, if the compression scheme is transferred to TD oscillators generating pulses with more than 60 MW peak power^22, 23^, high photon-flux XUV sources can be realized^40^. Eventually, with the ability to carrier-envelope-phase stabilize Kerr-lens mode-locked TD oscillators^44, 53^, compact XUV frequency comb or even MHz attosecond pulse sources are envisioned.

## Methods

BBO crystals were chosen because they are available at excellent commercial grade from multiple suppliers and they combine high damage threshold with reasonable nonlinearity. The negative uniaxial crystals were cut at the angles *θ* = 23.5° and *ϕ* = 90°. This corresponds to the phase-matching angle for SHG of 1030 nm with nearly maximized quadratic nonlinearity. By rotating the crystal, the phase-matching angle *θ* was tuned to about 21.5° which resulted in^30, 54, 55^:

2 $${n}_{2}^{({\rm{cas}})}=-\,\frac{4\pi }{{\varepsilon }_{0}{c}_{0}{\lambda }_{F}}\frac{{d}_{{\rm{eff}}}^{2}}{{n}_{SH}{n}_{F}^{2}{\rm{\Delta }}k}\approx -\,1.2\cdot {10}^{-15}\frac{{{\rm{cm}}}^{2}}{{\rm{W}}}.$$

3 $${\rm{\Delta }}k=\frac{4\pi }{{\lambda }_{F}}({n}_{SH}-{n}_{F})\approx 11.7\pi /{\rm{mm}}.$$

The vacuum permittivity is denoted by *ε*_0_, *c*_0_ is the speed of light in vacuum, *λ*_*F*_ = 1030 nm the wavelength of the fundamental, *d*_eff_ ≈ −2 pm/V the effective *χ* ^(2)^-nonlinearity, *n*_*SH*_ = 1.658 and *n*_*F*_ = 1.655 the refractive indices of the second harmonic and the fundamental, resp. Finally, Δ*k* denotes the phase-mismatch per unit length. The magnitude of the effective nonlinear refractive index induced by cascaded *χ* ^(2)^ processes, ${n}_{2}^{({\rm{cas}})}$, is about a factor of two higher than the Kerr nonlinearity of BBO at 1030 nm^56^. Additional graphs on magnitude and dispersion of the effected nonlinear index are provided in supplement 1.

The collimated beam diameters were about 1.8 mm, 1.8 mm and 2.4 mm in front of the first, second and third broadening stages, respectively. The focal lengths of the focusing lenses were 60 mm, 50 mm and 100 mm while *z*_min_ ≈ −10 mm in all stages. Due to the nonlinear defocusing, the waist sizes could not be measured directly. According to the simulations presented in Fig. 3, it is expected that peak intensities of about 180 GW/cm^2^ were reached inside the first crystal at full power. Due to the higher peak powers and similar focusing geometries in second and third stage, the peak intensities have been increased correspondingly for the shorter pulses. The pulse compression after the first spectral broadening stage was accomplished by means of highly dispersive mirrors. The semiconductors ZnSe, ZnS and TGG were also tested to compensate the down-chirp of the pulses but they adversely affected the beam profile at high average power. After the second spectral broadening stage, the dielectric material sapphire was used for pulse compression. In this case, no beam distortions were observed. Finally, the length of the third BBO crystal was chosen such that the pulses were fairly self-compressed when they emerged from the compression chamber.

The compressed pulses were measured with the second harmonic FROG described in ref. 28. The oscillator pulse duration was measured with a commercial autocorrelator. All mentioned pulse durations refer to the FWHM of the temporal intensity. The *M* ^2^ measurements were performed with a WinCamD *M* ^2^ stage.

The spatial grid of the simulations was set to 128 × 65 points with a size of 5 *μ*m × 5 *μ*m. Half of the x-y plane was simulated. The temporal grid had 512 points with 5 fs spacing and the centre frequencies near the fundamental (300 THz) and the second harmonic (600 THz) were factored out. The waves are propagated in frequency domain, and hence the simulations implicitly include self-steepening effects that arise from *χ* ^(2)^ and *χ* ^(3)^ effects^57^. The refractive index of BBO was derived from the material’s Sellmeier equation^55^. The Kerr nonlinearity was assumed to be isotropic and was set to ${n}_{2}^{({\rm{Kerr}})}$ = 4 · 10^−16^ cm^2^/W if not explicitly stated differently. Literature values, however, vary between 4 and 7 · 10^−16^ cm^2^/W^56^. The simulations included the mismatched second harmonic beam. Consequently, ${n}_{2}^{({\rm{cas}})}$ was a result of quadratic nonlinearities and not a parameter to the model as it could be guessed from Eq. (1).

## Electronic supplementary material

Supplementary Information
