# Supplementary material for: Efficient High-Power Ultrashort Pulse Compression in Self-Defocusing Bulk Media
Source: Sci Rep. 2017 May 3;7:1410. doi: 10.1038/s41598-017-01504-x (PMC5431174; doi:10.1038/s41598-017-01504-x)
Supplement: Supplementary file 1 — Supplementary Information [file 41598_2017_1504_MOESM1_ESM.pdf]

Supplementary Information:
Efficient High-Power Ultrashort Pulse Compression in Self-Defocusing Bulk Media

Marcus Seidel[1,2,*], Jonathan Brons[2,3], Gunnar Arisholm[4], Kilian Fritsch[2,3], Vladimir Pervak[2,3], and Oleg Pronin[1,2]

[1]Max-Planck-Institut für Quantenoptik, Hans-Kopfermann-Str. 1, D-85748 Garching, Germany
[2]UltraFast Innovations GmbH, Am Coulombwall 1, D-85748 Garching, Germany
[3]Ludwig-Maximilians-Universität München, Am Coulombwall 1, D-85748 Garching, Germany
[4]FFI (Norwegian Defence Research Establishment), P. O. Box 25, NO-2027 Kjeller, Norway
[*]marcus.seidel@mpq.mpg.de

Supplement 1: Magnitude and dispersion of the effective nonlinear refractive index

Fig. S1 displays magnitude and dispersion of the effective nonlinear refractive index for phase-mismatched quadratic nonlinearities near the type I second harmonic resonance. Fig. S1(a), which is calculated from the Sellmeier equations in ref. [S1], shows where this resonance occurs, i.e. for which tuning angle θ the SHG process is phase-matched, and indicates the regions of positive and negative $n_2^{(\mathrm{cas})}$. The curves in Figs. S1(b) and (c) have been calculated from Eq. (2) of the main text and a constant $n_2^{(\mathrm{Kerr})} = 4 \cdot 10^{-16}\mathrm{cm}^2/\mathrm{W}$. Fig. S1(b) demonstrates that BBO crystals must be tuned near resonance to achieve a defocusing nonlinearity at 1030 nm which implies dispersion of the n_2 as Fig. S1(c) illustrates.

Supplement 2: FROG measurements

Fig. S2 shows the measured and the retrieved FROG traces after the third broadening stage. The retrieval grid was 512 × 512, the delay spacing 2 fs and the wavelength spacing 0.26 nm. The FROG error of the retrieval was 0.3 %. The homogeneity of the beam is evidenced by the excellent agreement between the FROG and OSA spectrum. Since the FROG contains an only 10 μm thick BBO crystal for SHG, it behaves like a spatial filter[S2]. Moreover, Fig. S2(d) shows that additional control of the phase of the output pulses would lead to a further increase of peak power from 60 MW to 75 MW. The simulation utilized the FROG retrieval shown in Fig. S2(b) and added the GDD of the tailored mirrors used in Kerr-effect-based bulk broadening experiments presented in ref. [S3].

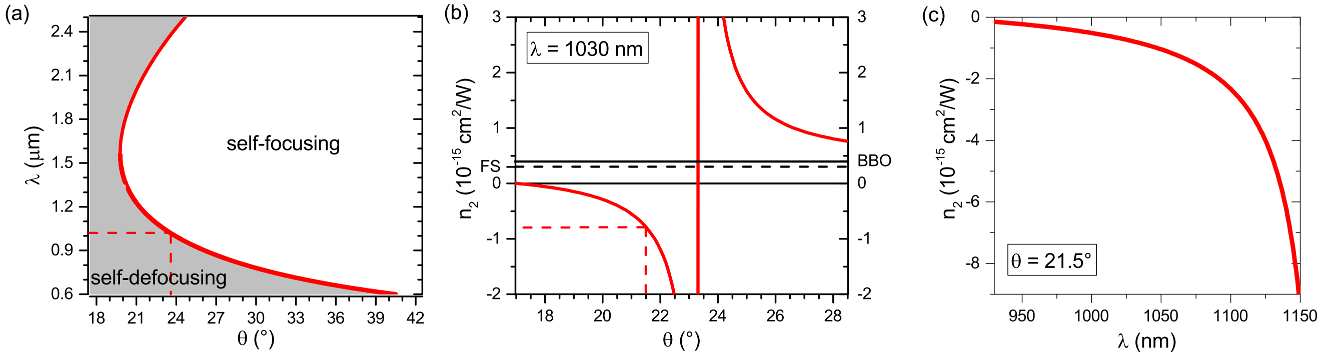

Figure S1. **(a)** Type I phase-matching curve for BBO. The dashed line shows the phase-matching angle, $\theta = 23.5°$, for 1030 nm, the pump wavelength. It corresponds to the angle the utilized BBOs were cut at. The grey-shaded area implies a negative $n_2^{(\mathrm{cas})}$. **(b)** The effective nonlinear refractive index of BBO for 1030 nm upon phase-mismatching. The red dashed line shows the angle at which the BBOs were operated. It corresponds to $n_2 = -8 \cdot 10^{-16}\mathrm{cm}^2/\mathrm{W}$. For comparison, $n_2^{(\mathrm{Kerr})}$ of BBO and fused silica (FS) are indicated. **(c)** The calculated dispersion of the effective nonlinear refractive index for the tuning angle $\theta = 21.5°$.

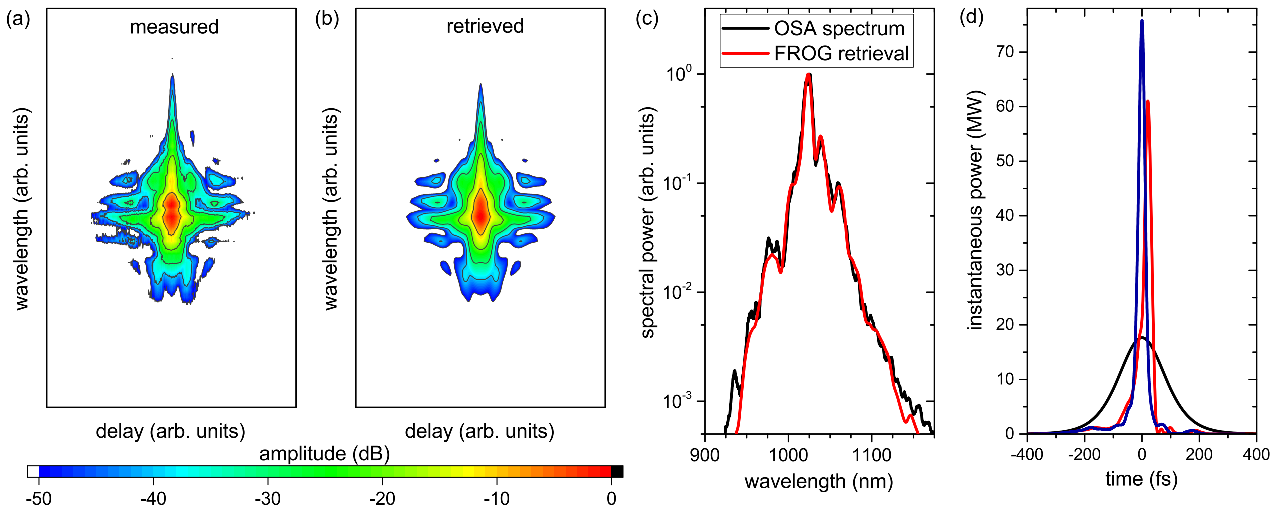

Figure S2. **(a)** measured and **(b)** retrieved FROG traces of the compressed pulse after the third broadening stage. The scale is logarithmic. **(c)** Spectrum measured with an optical spectrum analyser compared to the retrieved FROG spectrum of the trace from (b). The scale is also logarithmic. **(d)** Instantaneous power of the 90 W average power oscillator output pulses (black line), the 70 W average power compressed pulses after the third stage (red line) and the simulated pulses which were compressed by two bounces off chirped mirrors with tailored GDD (blue line).

Supplement 3: Ray model for self-focusing and self-defocusing nonlinearities

In order to visualize the homogenization effect upon combining self-defocusing nonlinearities with beam convergence, the simple model proposed by R.Y. Chiao et *al.* [S4,S5] is adapted. The initial model was used to find a threshold for self-trapping of optical beams. It treated the laser beam as a step-index fibre-like waveguide with a cladding of refractive index n and a core of refractive index $n + n_2 I$ where I is the laser intensity. This led to a good approximation of a medium's critical power. Here, the laser beam is treated as a graded-index waveguide with the refractive index profile

$$n(r) = n + n_2 I_0 \exp\left\{-2r^2/w^2\right\}, \tag{1}$$

where I_0 is the peak intensity, r the distance to the beam centre and w the beam radius at the front facet of the nonlinear crystal. Typically, ray tracing in graded index optical elements is performed by applying Fermat's principle. For cylindrical symmetry, this leads to the equation

$$\frac{d^2r}{dz^2} = \frac{1}{n(r)}\frac{dn(r)}{dr} \approx \frac{1}{n}\frac{dn(r)}{dr} = -\frac{4n_2 I_0}{w^2 n} r \exp\left\{-2r^2/w^2\right\}, \tag{2}$$

where z is the propagation length inside the crystal. The differential equation was solved numerically with a Runge-Kutta method. It is to note that no z-dependence was assigned to I_0 and w which is a strong approximation since the beam size is changing inside the crystal.

Ray packet equivalents (consiting of 100 rays each) of three Gaussian beams [S6] were launched. Their propagation through a 10 mm long nonlinear crystal was plotted in Fig. S3. Fig. S3(a) presents the modelling of the self-focusing example discussed in the main text (cf. Fig. 3(e)). The laser beam is focused to a waist of 20 μm. In analogy to the simulation presented in the main text, the BBO crystal with $n_2 = +8 \cdot 10^{-16}\text{cm}^2/\text{W}$ is placed at $z_{\text{min}} = 4z_R$. The rays which are initially in the beam centre (red rays) remain in this most intense region due to the self-focusing effect. Therefore, they undergo strong SPM while the rays which are initially in the wing of the beam (black rays) hardly notice the self-focusing effect, diverge strongly, and thus do not undergo SPM. This behaviour is in good agreement with the simulation result shown in Fig. 3(e) of the main text. The central part of the beam is spectrally broadened while the spectrum of the wings is not affected by the nonlinearity of the crystal. The plot of Fig. S3(b) corresponds to the simulation result shown in Fig. 3(c) of the main text. The n_2 is set to $-8 \cdot 10^{-16}\text{cm}^2/\text{W}$ and $z_{\text{min}} = -7z_R$. The rays behave fundamentally different from the self-focusing example. The initially central rays are strongly diffracted and cross the rays from the wings within the defocusing medium, i.e. they undergo SPM only at the beginning of the crystal while the rays from the wings will spectrally broaden towards the end of the crystal. This leads to the homogenization effect in the case of combining self-defocusing and beam convergence. Finally, the case of self-defocusing without divergence is studied in Fig. S3(c). The peak intensity is set to 200 GW/cm^2 and $n_2 = -8 \cdot 10^{-16}\text{cm}^2/\text{W}$ like before. Fewer rays cross

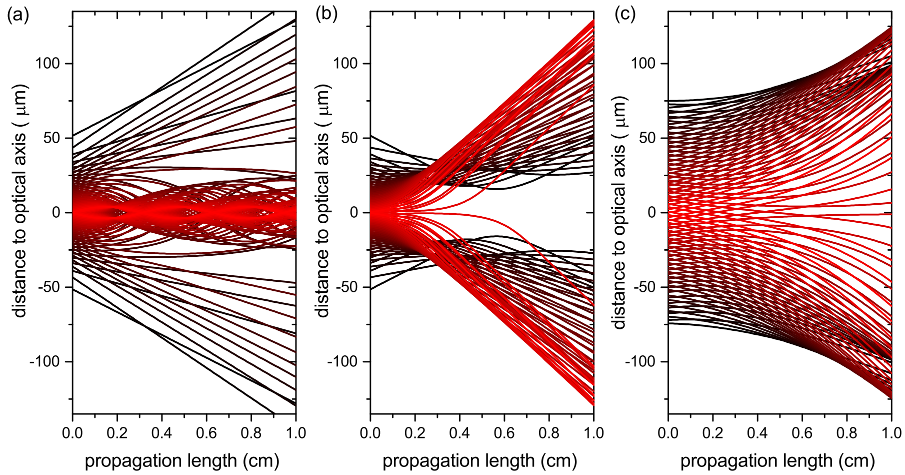

Figure S3. Model for 100 rays describing a Gaussian beam. The darker the rays the longer the initial distance to the optical axis. **(a)** Self-focusing nonlinearity and beam divergence: The central rays stay close to the optical axis while the outer rays diverge strongly. **(b)** Self-defocusing nonlinearity and beam convergence: The inner rays are diffracted out of the inner beam region while the outer rays move towards the beam centre. **(c)** Self-defocusing nonlinearity, no divergence: the mixing of outer and inner rays is clearly reduced in comparison to (b). For all cases, an initial peak intensity of 200 GW/cm^2 was assumed.

---

within the crystal. Basically, only the initially centred rays undergo SPM in the beginning of the nonlinear medium. The rays from the wings diffract slower but they do not propagate towards the beam centre due to the missing convergence. In conclusion, the simple ray model provides an illustrative picture of the homogenization effect which was quantitatively investigated in the main text.

Supplement 4: Spectral broadening in dependence on tuning angle and distance to the focal point

In order to investigate the sensitivity of the pulse compression scheme on the alignment of the BBO crystals, simulations with different phase-matching angles and varied crystal positions have been conducted. The simulations refer to the first broadening stage. The results are shown in Fig. S4.

In Fig. S4(a) the tuning angle is fixed to $\theta = 21.5°$, i.e. the effective n_2 is approximately $-8 \cdot 10^{-16}\,\mathrm{cm}^2/\mathrm{W}$. The crystal length is 10 mm which corresponds to about 8 Rayleigh lengths. If the n_2 was 0, the maximal peak intensity inside the crystal would be about 3 TW/cm^2 for z_{min} between -8 and 0 z_R. This would lead to crystal damage. It is to note that the critical intensity

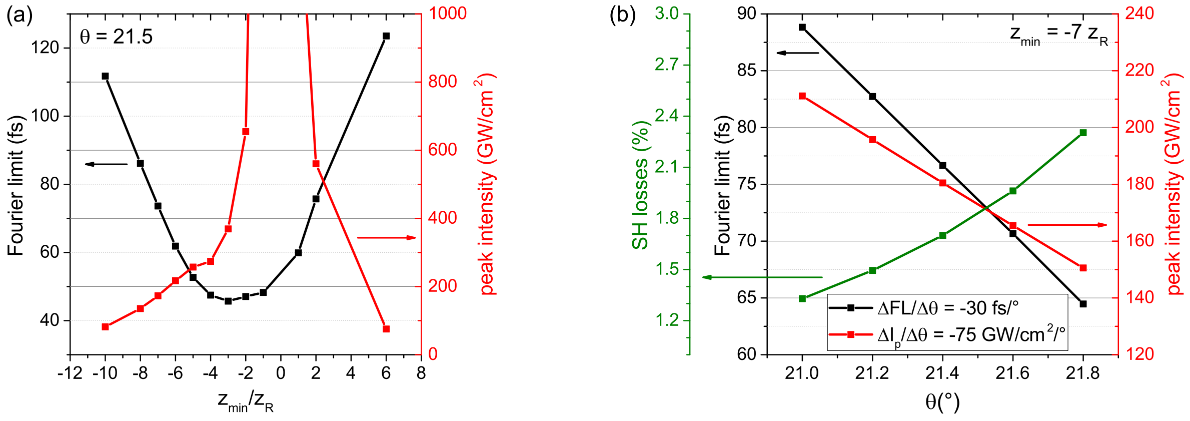

Figure S4. **(a)** Dependence of the Fourier limit and the maximal peak intensity inside the 10 mm long crystal on z_{min}. The Rayleigh length z_R is 1.22 mm for a beam waist of 20 μm. The tuning angle θ is fixed to 21.5°. **(b)** Dependence of the Fourier limit, the maximal peak intensity inside the crystal and the second harmonic (SH) losses on the tuning angle θ for a fixed $z_{\mathrm{min}} = -7\,z_R$.

has not been measured for the oscillator parameters. For BBO, it is specified to be 40 GW/cm^2 for tens of ps pulse duration[S7] while tens of TW/cm^2 did not damage the crystal when 4 fs pulses were used[S8]. For the utilized high-power fs system, a damage threshold of several hundreds of GW/cm^2 is estimated. Crystal damage was observed upon detuning of the crystal angle by a few degrees. The self-defocusing effect strongly reduces the maximal peak intensity inside the crystal to only about 6 % of the free-space value if $z_{\min}$ = - 7 z_R. This, however, depends on the tuning angle as Fig. S4(b) shows. The slope of the maximal peak intensity in dependence on the tuning angle is about -75 GW/cm^2/° in the vicinity of $\theta = 21.5°$, i.e. the angle has to be set carefully before power is sent into the broadening crystal in order to avoid its damage. The generated second harmonic provides a good measure for the tuning angle. For the 10 mm crystal about 2 % (1.8 W) of the 90 W are converted to green light. The Fourier limit of the broadened pulses is also very sensitive to the tuning angle as Fig. S4(b) indicates. In the vicinity of $\theta = 21.5°$, the slope is -30 fs/°. It is remarkable that the peak intensity grows with the Fourier limit. This is due to the tuning angle dependence of n_2. An increase in θ leads to higher absolute value of the nonlinearity, subsequently to a lower Fourier limit as well as to stronger self-defocusing, and thus to a reduced maximal peak intensity inside the crystal. Peak intensity and the broadening factor are also strongly dependent on the crystal position with respect to the focal point. In the graph shown in Fig. S4(a), crystal damage is expected if $z_{\min}$ is in the interval between $\approx \pm 4$ Rayleigh lengths. If the crystal is positioned behind the focus, the broadening factor is relatively low. In the example, the best positions for operation are between -8 and -4 z_R where a Fourier limit of sub-100 fs is reached and the peak intensity is kept below the damage threshold. As described in the main text, $z_{\min} \approx -10$ mm was chosen which enabled a relatively clean pulse compression by compensating for first order chirp only. The initial experimental setup required a careful choice of nonlinearity and divergence as described in this section. The presented simulations helped finding an appropriate setting. After having established the reported configuration, the setup has been operated for several months on a day-to-day basis without the need of major changes.

References

S1. Zhang, D., Kong, Y. & Zhang, J.-y. Optical parametric properties of 532-nm-pumped beta-barium-borate near the infrared absorption edge. *Optics Communications* **184**, 485 – 491 (2000).

S2. Seidel, M., Arisholm, G., Brons, J., Pervak, V. & Pronin, O. All solid-state spectral broadening: an average and peak power scalable method for compression of ultrashort pulses. *Opt. Express* **24**, 9412–9428 (2016).

S3. Pronin, O. *et al.* High-power multi-megahertz source of waveform-stabilized few-cycle light. *Nat. Commun.* **6**, 6988 (2015).

S4. Chiao, R. Y., Garmire, E. & Townes, C. H. Self-Trapping of Optical Beams. *Phys. Rev. Lett.* **13**, 479–482 (1964).

S5. Boyd, R. W. Chapter 7 - Processes Resulting from the Intensity-Dependent Refractive Index. In Boyd, R. W. (ed.) *Nonlinear Optics*, 329 – 390 (Academic, Burlington, 2008), third edn.

S6. Steier, W. H. The Ray Packet Equivalent of a Gaussian Light Beam. *Appl. Opt.* **5**, 1229–1233 (1966).

S7. Li, H., Zhou, F., Zhang, X. & Ji, W. Bound electronic Kerr effect and self-focusing induced damage in second-harmonic-generation crystals. *Optics Communications* **144**, 75 – 81 (1997).

S8. Fattahi, H., Schwarz, A., Keiber, S. & Karpowicz, N. Efficient octave-spanning difference-frequency generation using few-cycle pulses in simple collinear geometry. *Opt. Lett.* **38**, 4216–4219 (2013).

(URLs accessed on 03/23/2017)
